# Real-Time Temperature Monitoring under Thermal Cycling Loading with Optical Fiber Sensor

**DOI:** 10.3390/s22124466

**Published:** 2022-06-13

**Authors:** Shiuh-Chuan Her, Jr-Luen Tasi

**Affiliations:** Department of Mechanical Engineering, Yuan Ze University, Chung-Li 320, Taoyuan 32003, Taiwan; s985033@mail.yzu.edu.tw

**Keywords:** fiber Bragg grating, optical band pass filter, thermal cycling loading, real-time

## Abstract

A fiber optic sensing system consisting of a fiber Bragg grating (FBG) sensor, optical circulator, optical band pass filter and photodetector is developed to monitor the real-time temperature response of a structure under a dynamic thermal loading. The FBG sensor is surface-bonded on a test specimen and integrated with an optical band pass filter. As a broadband light source transmits into a FBG sensor, a specific wavelength is reflected and transmitted into an optical band pass filter. The reflected wavelength is significantly affected by the temperature, while the output light power from the optical band pass filter is dependent on the wavelength. By measuring the light power with a photodetector, the wavelength can be demodulated, resulting in the determination of the temperature. In this work, the proposed optical sensing system was utilized to monitor the dynamic temperature change of a steel beam under a thermal cycling loading. To verify the accuracy of the fiber optic sensor, a thermocouple was adopted as the reference. The experimental results illustrate a good agreement between the fiber optic sensor and thermocouple. Electronic packages composed of various components such as a solder joint, silicon die, mold compound, and solder mask are frequently subjected to a thermal cycling loading in real-life applications. Temperature variations’ incorporation with mismatches of coefficients of thermal expansion among the assembly components leads to crack growth, damage accumulation and final failure. It is important to monitor the temperature to prevent a thermal fatigue failure. A fast response and easy implementation of the fiber optic sensing system was proposed for the real-time temperature measurement under thermal cycling loading.

## 1. Introduction

Sensing technology plays an important role in structural health monitoring, since in-service monitoring can provide real-time information to improve the safety and performance of engineering structures. Various sensors with different working principles have been proposed for structural health monitoring [1,2]. Traditional strain or temperature sensors such as a strain gauge and thermocouple mainly rely on electrical working principles to detect strain or temperature. The major drawbacks of these sensors include the sensitivity, cross talk, vulnerability to harsh environmental conditions and their sizes [3]. Fiber optic sensors have several advantages over traditional sensors, such as a compact size, corrosion resistance, multiplex capability, and immunity to electromagnetic interference [4,5]. Moreover, fiber optic sensor exhibits a broad bandwidth, which permits a fast transmission of a huge amount of information in the same optical fiber [6]. With the rapid progress of optical fiber sensing technologies, many fiber optic sensors based on different operational principles have been developed, such as reflectometry-based sensors (Brillion and Rayleigh scattering), grating-based sensors (FBG), and interferometry-based sensors (Mach–Zehnder and Michelson) [7,8]. Choi et al. [9] detected the impact location and residual strain of a composite pressure vessel using a phase-modulated optical fiber sensor based on Brillouin frequency. Lu et al. [10] developed a distributed optical fiber sensing system to discriminate temperature and strain based on coherent Rayleigh scattering. Zhao et al. [11] proposed a low-frequency vibration sensor based on the Mach–Zehnder Interferometer with a maximum error of 0.27%. Li et al. [12] developed a highly sensitive optical sensor based on the Michelson interferometer with a measured sensitivity of up to 77 pm/mm for a water level ranging from 0 to 40 mm.

FBG sensors based on the shift of the reflecting particular wavelength of light are the most common fiber sensing technology for structural health monitoring (SHM) [13]. They are highly sensitive to changes in both the strain and temperature. Due to the mature processing technology and low fabrication cost, a variety of applications for FBG sensors have been proposed. Szebenyi et al. [14] demonstrated the capability of FBG sensors integrated between the reinforcing layers of composites for cyclic creep strain monitoring and fatigue failure prediction. Hong–kun et al. [15] reported an optical fiber pressure sensor using two FBGs to reduce the cross-sensitivity between the temperature and pressure, leading to a high pressure sensitivity of 1.198 nm/MPa in the range of 0–1 MPa. Lv et al. [16] developed an optical fiber sensor consisting of two FBGs for simultaneous measurements of the flow rate and temperature in a pipeline, with an accuracy of 2.27% and ±1 °C, respectively. Jang and Kim [17] utilized an FBG sensor to detect impact-generated acoustic emission (AE) signals and to analyze the wavelet transform (WT) method for real-time delamination monitoring of a composite plate. Weisz–Patrault et al. [18] evaluated the contact stress during a cold rolling process with FBG sensors. Tsukada et al. [19] investigated the residual strain distribution in a thick thermoplastic composite during a high-rate manufacturing process using FBG sensors. Anastasopoulos et al. [20] developed a novel damage detection method based on the strain modal shape obtained from fiber-optic Bragg grating strain sensors.

Temperature sensors have been widely used for temperature monitoring in many different applications such as machinery components, power plants, electric devices, kitchen appliances, heating ventilation and air conditioning devices. There are many different types of temperature sensors, based on different working principles, that are used to measure the temperature. Among them, thermocouples and thermistors are the most common temperature sensors that are used. Both thermocouples and thermistors are point-wise sensors, and their lacking capability in terms of continuum-wise measurements is their major drawback. On the contrary, FBG sensors are highly sensitive to the temperature, and their multiplex capability can be prepared in a series of same optical fibers in order to conduct continuum-wise measurements. In this work, an FBG sensor is incorporated with an optical band pass filter and a photodetector to monitor the temperature of structures. The wavelength shift of the FBG sensor induced by a temperature change is converted to a variation of light power through an optical band pass filter. The light power is measured by a photodetector. A fast response of the photodetector allows for the real-time monitoring of the structural temperature. Thermal cycling is an environmental test that is commonly used to evaluate the reliability of materials or electronic devices and to detect any manufacturing defects early during the thermal fatigue. Electronic packages composed of various components such as a solder joint, silicon die, mold compound, and solder mask are frequently subjected to a thermal cycling loading in real-life applications [21]. Temperature variations’ incorporation with mismatches of coefficients of thermal expansion among the assembly components leads to crack growth, damage accumulation and final failure [22]. It is important to monitor the temperature to prevent a thermal fatigue failure. The proposed FBG sensor was employed in order to monitor the real-time temperature response under a thermal cycling loading.

## 2. Bragg Wavelength Shift

FBG possesses a periodic variation in the core refractive index along a length of an optical fiber. When a broadband light transmits into FBG, a specific wavelength known as Bragg wavelength $\lambda_{B}$ is reflected, while the remaining portion of the broadband light passes through. The Bragg wavelength can be expressed in terms of the grating period Λ and refractive index $n_{0}$ as follows:

(1) $$\lambda_{B}=2n_{0}\Lambda$$

The changes of the grating period and refractive index due to the mechanical strain ε and temperature variation ΔT exerted on the FBG induce a Bragg wavelength shift $\Delta \lambda_{B}$, which can be expressed in terms of the sum of the strain and temperature contributions as follows [23]:

(2) $$\frac{\Delta \lambda_{B}}{\lambda_{B}}=k_{\varepsilon }\varepsilon +k_{T}\Delta T$$

(3) $$k_{\varepsilon }=1-\frac{1}{2}n_{0}^{2}\left[\left(1-\upsilon_{f}\right)p_{12}-\upsilon_{f}p_{11}\right]$$

(4) $$k_{T}=\left[1-\frac{1}{2}n_{0}^{2}\left(p_{11}+2p_{12}\right)\right]\alpha_{f}+\frac{\xi }{n_{0}}$$

where $k_{T}$ and $k_{\varepsilon }$ represent the temperature and strain coefficients of the FBG sensor, respectively, $n_{0},\;\upsilon_{f},\;\alpha_{f}\;\mathrm{and}\;\xi$ are the refractive index, Poisson’s ratio, coefficient of thermal expansion and thermo-optic coefficient of the optical fiber, respectively, and $p_{11}\;\mathrm{and}\;p_{12}$ denote Pockel’s constants.

The Bragg wavelength shift of an FBG sensor adhering to a host structure due to a temperature change can be attributed to the temperature change Δ*T* and the thermal strain $\varepsilon_{T}$ induced by the mismatch of the thermal expansion between the optical fiber and host structure. The thermal strain $\varepsilon_{T}$ has been reported by Her and Huang [24]. Substituting the temperature change Δ*T* and thermal strain $\varepsilon_{T}$ into Equation (2) leads to the determination of the Bragg wavelength shift of an FBG sensor as follows:

(5) $$\Delta \lambda_{B}=\left\{1-\frac{1}{2}n_{0}^{2}\left[\left(1-\upsilon_{f}\right)p_{12}-\upsilon_{f}p_{11}\right]\right\}\varepsilon_{T}\lambda_{B}$$

$$+\left\{\left[1-\frac{1}{2}n_{0}^{2}\left(p_{11}+2p_{12}\right)\right]\alpha_{f}+\frac{\xi }{n_{0}}\right\}\Delta T\lambda_{B}$$

**Figure 1:**
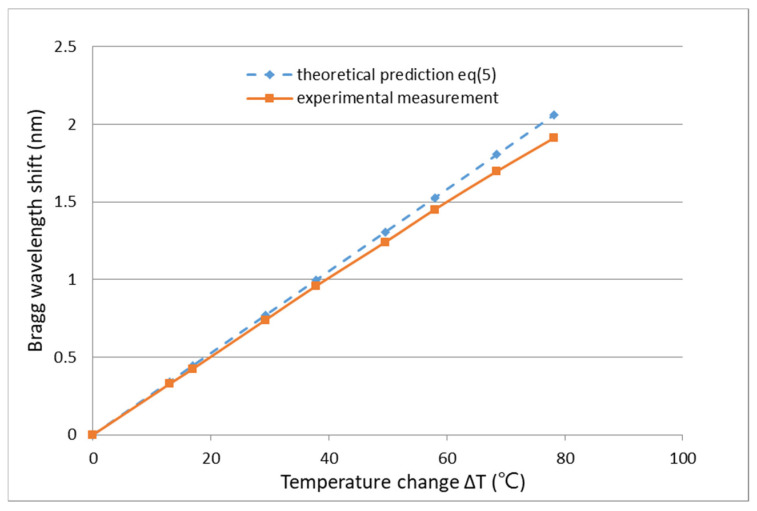
Bragg wavelength shift of a fiber Bragg grating (FBG) sensor induced by a temperature change.

In the experimental test, a steel beam bonded with an FBG sensor was placed in a thermal chamber and heated from room temperature to 100 °C. The Bragg wavelength shift of the surface-bonded FBG sensor due to the temperature change was detected by an optical spectrum analyzer (AQ 6331, Ando Electric Co., Tokyo, Japan). The experimental measurements of the Bragg wavelength shift were compared with the theoretical prediction of Equation (5), as illustrated in Figure 1. The grating temperature coefficient of 25 pm/°C extracted from Figure 1 is higher than that of the normal grating temperature coefficient of 10 pm/°C due to the thermal strain transferred from the host structure to the FBG sensor. The coefficients of the thermal expansion of the FBG sensor and steel host structure are $0.5\times 10^{-6}/{}^{\circ}C$ and $12\times 10^{-6}/{}^{\circ}C$, respectively. The mismatch of the thermal expansion between the optical fiber and host structure induces a thermal strain leading to an additional Bragg wavelength shift. The theoretical prediction is in good agreement with the experimental measurement. A linear dependence between the Bragg wavelength shift and temperature change was observed.

## 3. Temperature-IEEEnduced Light Power Variation

**Figure 2:**
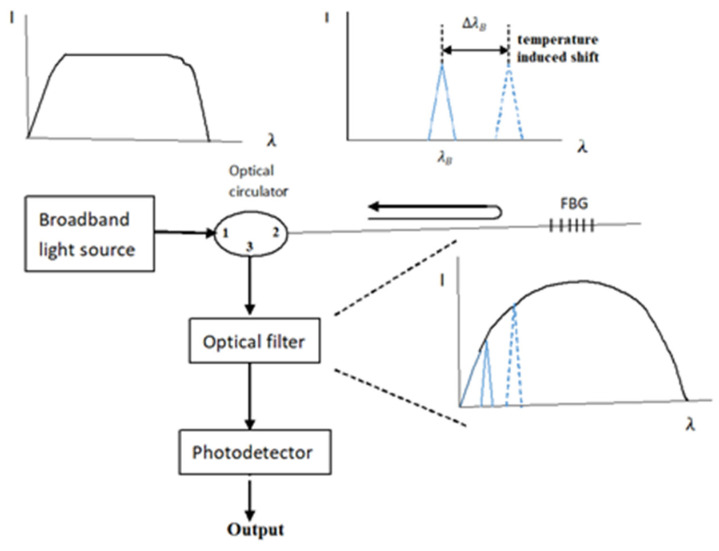
Schematic diagram of the FBG sensing system [25].

**Figure 3:**
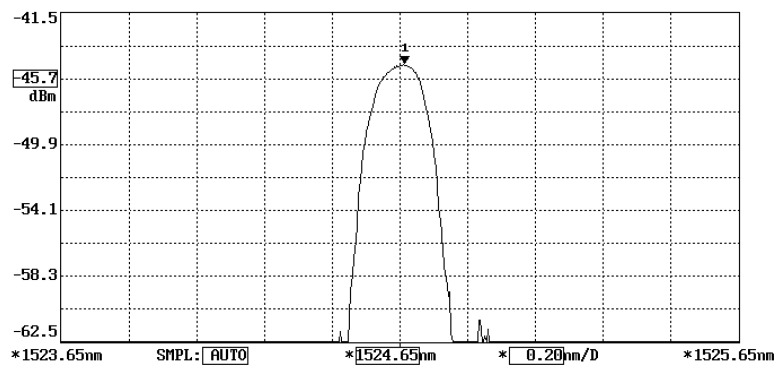
Bragg wavelength reflected from the FBG sensor.

**Figure 4:**
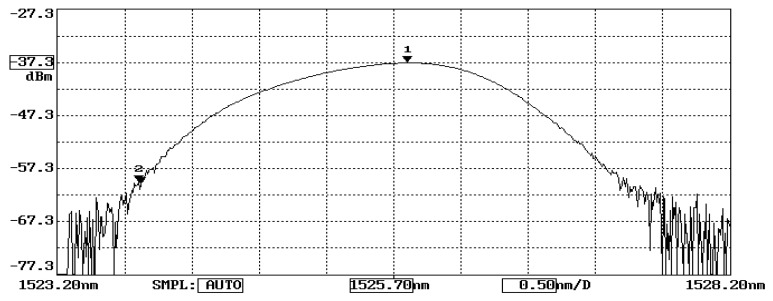
Band pass from the optical filter.

The working principle of FBG sensors exists according to the correlation between the Bragg wavelength shift and the temperature change and applied strain, as presented in Equation (2). A wavelength meter is widely employed to measure the wavelength. However, a slow response of the wavelength meter is inappropriate for online and real-time measurements. In this study, the Bragg wavelength shift is converted to a modulation of light power through an optical band pass filter and detected by a photodetector. The fast response of a photodetector provides the feasibility of the measurement for real-time responses. The proposed FBG sensing system is schematically illustrated in Figure 2. The sensing system is composed of a broadband light source (ASE 1550A, Faztec Optronics Corp., Taipei, Taiwan), an optical circulator, an optical band pass filter (TBF-1550-1.0, Newport Corp., Irvine, CA, USA) and a photodetector (ET3010, Throlabs Inc., Newton, NJ, USA). The initial Bragg wavelength of the FBG sensor used in this study is 1524.66 nm, as shown in Figure 3, while the range of the optical band pass filter is 1523.8~1527.2 nm, as shown in Figure 4. When a broadband light is transmitted to the FBG, a narrowband spectrum of the Bragg wavelength is reflected back from the FBG sensor and guided into an optical band pass filter. The light power is modulated by the optical band pass filter according to its wavelength. To ensure the one-to-one correspondence between the wavelength and light power, the Bragg wavelength reflected from the FBG sensor was restricted to a range of 1523.8~1525.7 nm in this study. In this wavelength region, the output of the light power from the optical band pass filter increases with the increase of the wavelength. Thus, a one-to-one correspondence between the temperature and light power can be achieved. In this work, a Bragg wavelength of 1524.66 nm was used for the FBG sensor since it falls in the valid region of 1523.8~1525.7 nm. The light power is converted to an electrical voltage through a photodetector, yielding to the determination of the temperature.

**Figure 5:**
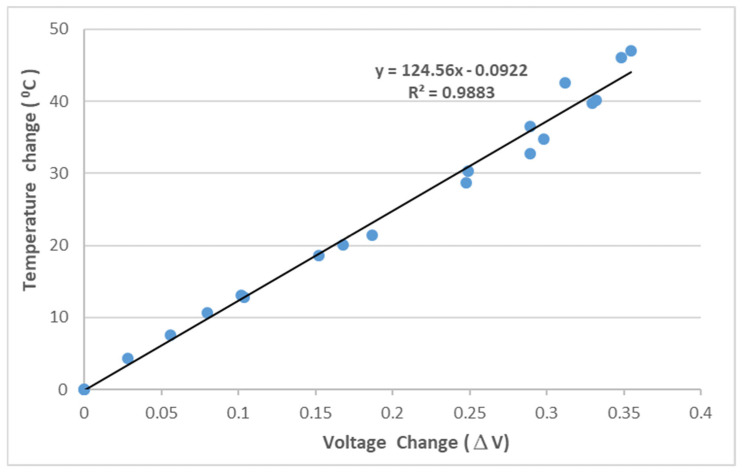
Relationship between the temperature change and light power variation.

To correlate the temperature with the light power, experimental tests of an FBG sensor adhering to a steel beam under a series of temperature changes were conducted. The Bragg wavelength of the FBG sensor used in this study at its initial state is $\lambda_{B}=1524.66\;\mathrm{nm}$, as shown in Figure 3, which is in the left half region of the optical band pass filter, as shown in Figure 4. The output light power of the optical band pass filter increases with the increase of the temperature due to the increase of the Bragg wavelength reflected from the FBG sensor. In the experimental tests, the test specimen was heated from room temperature to 70 °C in a thermal chamber. The temperature change and light power change were recorded and plotted in Figure 5. The linear relationship fitting between the temperature change and light power variation exhibits a reasonable linearity, with the coefficient of determination being approximately 0.99. The linear relationship between the temperature change ΔT and light power variation Δ*V* is obtained as follows:

(6) ΔT=124.56ΔV

## 4. Thermal Cycling Test

**Figure 6:**
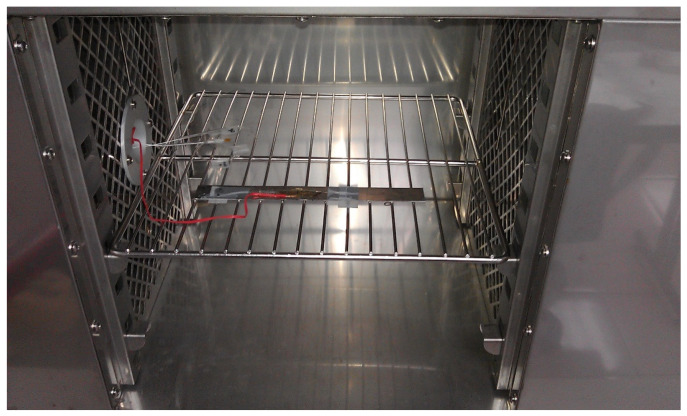
Experimental setup of a test specimen in a thermal cycling chamber.

**Figure 7:**
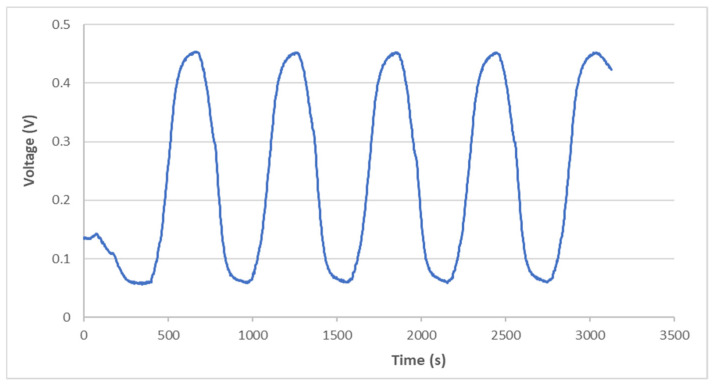
Variation of light power during the thermal cycling test with a heating rate of 10 °C/min.

**Figure 8:**
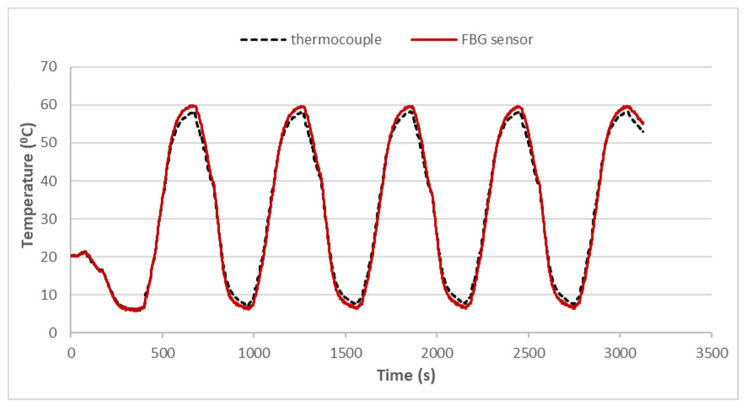
Dynamic temperature response of the specimen in the thermal cycling test with a heating rate of 10 °C/min.

**Figure 9:**
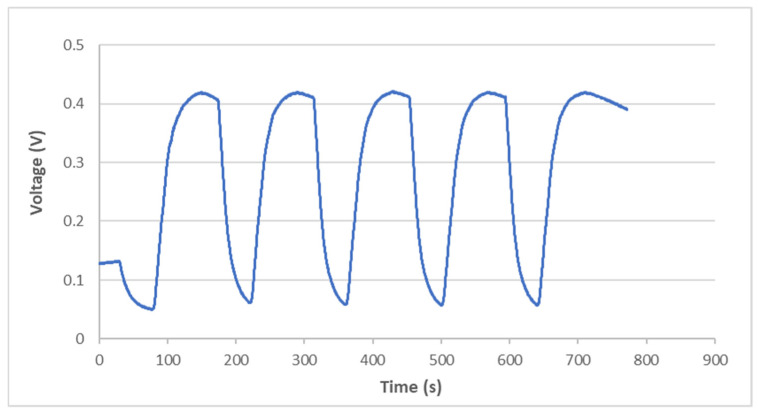
Variation of light power during the thermal cycling test with a heating rate of 40 °C/min.

**Figure 10:**
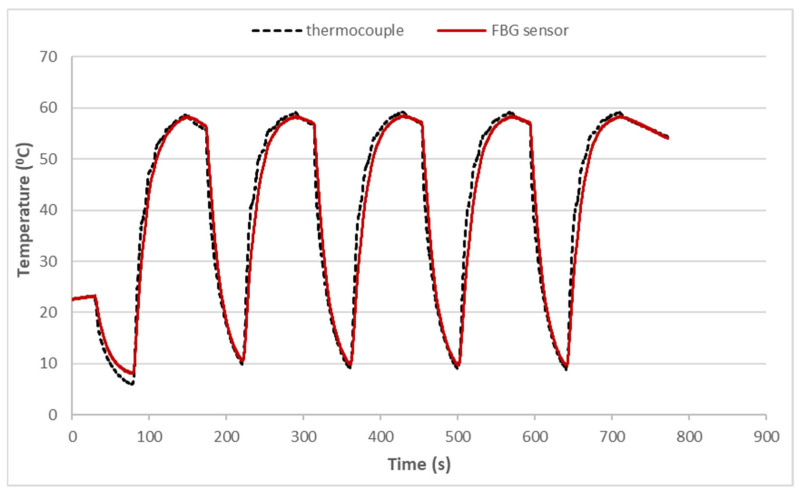
Dynamic temperature response of the specimen in the thermal cycling test with a heating rate of 40 °C/min.

A thermal cycling test is a process of temperature change at a high rate for materials or devices. It is considered to be an environmental stress test that can be used to evaluate the durability and safety of products. A thermal cycling test can quickly unravel the potential failure or defects of an electronic product to improve product design and reliability. In general, electronic devices are under a non-uniform strain distribution in a working environment. The non-uniform strain distribution of the electronic component could affect the Bragg wavelength shift of the attached FBG sensor. An FBG sensor with a small size can provide a point-wise measurement. Moreover, the non-uniformity within the small area is insignificant. In this work, an FBG sensor was employed to monitor the temperature change in a thermal cycling test. A steel beam surface-bonded FBG sensor, using epoxy and with a length of 2 cm, was placed in a thermal cycling test chamber (TSR-A4, KingSon Technology Co., New Taipei City, Taiwan), as shown in Figure 6. In addition, a K-type thermocouple with a diameter of 2 mm was placed next to the FBG sensor as a reference. Two different heating rates, 10 °C/min and 40 °C/min, were adopted in the thermal cycling test. The valid wavelength range for the optical band pass filter with a one-to-one correspondence between the wavelength and light power was 1523.8~1525.7 nm. The temperature variation was 50 °C in the experimental tests to make sure that the Bragg wavelength reflected from the FBG sensor was in the range of 1523.8~1525.7 nm. The light power detected by the photodetector in the thermal cycling test with a heating rate of 10 °C/min is plotted in Figure 7. The initial temperature $T_{0}$ and light power $V_{0}$ were 22.80 °C and 0.1268 V, respectively. Substituting the light power from Figure 7, the initial temperature $T_{0}$ and the light power $V_{0}$ into Equation (6), the dynamic temperature response of the specimen in the thermal cycling test can be detected, as shown in Figure 8. The temperature measured by the FBG sensor was compared with the results recorded by a thermocouple. It can be seen that a close agreement with a difference of less than 2% was achieved between the FBG sensor and thermocouple. Next, the heating rate was increased to 40 °C/min. Figure 9 and Figure 10 show the variation of light power and temperature response of the specimen during the thermal cycling test, respectively. It demonstrates that the FBG sensor is capable of monitoring the real-time temperature response at a high rate of 40 °C/min.

## 5. Conclusions

FBG sensors have been well recognized as being more suitable for structure health monitoring than conventional electrical sensors, with capabilities of immunity to electromagnetic interference and multiplexed measurements. In this study, a fiber optic sensing system consisting of an FBG sensor, optical band pass filter, and photodetector was developed to monitor the real-time temperature response of a structure subjected to a dynamic thermal loading. The shift of the Bragg wavelength reflected from the FBG sensor induced by the temperature change is converted to a light power variation through an optical band pass filter and detected by a photodetector. Through a fast response of the photodetector, in situ monitoring of dynamic temperature change is possible. The FBG sensing system was employed to conduct temperature monitoring during a thermal cycling loading with a high rate of 40 °C/min. The FBG measurement was verified by a thermocouple, with a difference of less than 2%. Moreover, the proposed optic fiber sensing system has the advantages of having a compact size, good accuracy, low cost and easy fabrication. The present work provides an FBG sensing system with a fast response, online monitoring and easy implementation, which is helpful and useful for structural health monitoring.

## Data Availability

Data are available on request.
